# Genetic Engineering of *Streptomyces ghanaensis* ATCC14672 for Improved Production of Moenomycins

**DOI:** 10.3390/microorganisms10010030

**Published:** 2021-12-24

**Authors:** Roman Makitrynskyy, Olga Tsypik, Andreas Bechthold

**Affiliations:** Department of Pharmaceutical Biology and Biotechnology, Institute of Pharmaceutical Sciences, Albert-Ludwigs University, 79104 Freiburg, Germany; olga.tsypik@pharmazie.uni-freiburg.de

**Keywords:** *Streptomyces*, moenomycin, natural products, AdpA, *oriC*, WblA, antibiotic, morphology, regulation

## Abstract

Streptomycetes are soil-dwelling multicellular microorganisms famous for their unprecedented ability to synthesize numerous bioactive natural products (NPs). In addition to their rich arsenal of secondary metabolites, *Streptomyces* are characterized by complex morphological differentiation. Mostly, industrial production of NPs is done by submerged fermentation, where streptomycetes grow as a vegetative mycelium forming pellets. Often, suboptimal growth peculiarities are the major bottleneck for industrial exploitation. In this work, we employed genetic engineering approaches to improve the production of moenomycins (Mm) in *Streptomyces ghanaensis*, the only known natural direct inhibitors of bacterial peptidoglycan glycosyltransferses. We showed that in vivo elimination of binding sites for the pleiotropic regulator AdpA in the *oriC* region strongly influences growth and positively correlates with Mm accumulation. Additionally, a marker- and “scar”-less deletion of *moeH5*, encoding an amidotransferase from the Mm gene cluster, significantly narrows down the Mm production spectrum. Strikingly, antibiotic titers were strongly enhanced by the elimination of the pleiotropic regulatory gene *wblA*, involved in the late steps of morphogenesis. Altogether, we generated Mm overproducers with optimized growth parameters, which are useful for further genome engineering and chemoenzymatic generation of novel Mm derivatives. Analogously, such a scheme can be applied to other *Streptomyces* spp.

## 1. Introduction

The members of the genus *Streptomyces* are well known as one of the richest sources of biologically active secondary metabolites of microbial origin [1]. Because of their saprophytic lifestyle, streptomycetes are also able to decompose plenty of naturally occurring polymers by producing and exporting various hydrolytic enzymes outside the cells. Some of those enzymes are of high industrial interest, such as cellulases, proteases and amylases [2]. These unique attributes explain the intensive research and strong industrial interest in *Streptomyces* biology.

In contrast to most bacteria, streptomycetes are multicellular microorganisms, which are also notable for their complex morphological differentiation [3]. Normally, their life cycle begins from a spore, which germinates in the germ tubes, encountering favorable conditions and nutrients. These germ tubes grow by tip extension, forming thread-like structures named hyphae. New hyphae develop via subapical branching, leading to the formation of a dense complex of interconnected hyphae called a vegetative mycelium. In response to certain signals, such as nutrient depletion, morphological differentiation is initiated in a form of long non-fragmented aerial hyphae, which rise from the vegetative mycelium. The entire life cycle culminates with a regular septation of aerial hyphae in separate compartments, which ultimately develop into long chains of mature spores [4].

In a submerged environment streptomycetes typically grow in a form of vegetative mycelium, predominantly forming dense particles or clumps [5]. In contrast to unicellular bacteria such as *Escherichia coli* and *Bacillus* spp., the *Streptomyces* growth type is less attractive from a biotechnological point of view, for several reasons. Particularly, such growth results in mass- and heat-transfer problems, and in oxidative stress [6,7]. These consequentially lead to slow growth rates, highly viscous cultures and high culture heterogeneity [8]. Large mycelial clumps are mainly physiologically active around the edge of the pellet, with a significant restriction of the effective transfer of nutritionals and gases to its center [9]. Formation of pellets is a complex process, which includes two major factors—aggregation of germinating spores and pellet fragmentation—influencing the final pellet size heterogeneity. Aggregation is driven by glycans associated with cellular surface, encoded by the *cslA/glxA* operon and the *mat* cluster [10,11,12], whereas pellet fragmentation consists of the detachment of mycelial portions from pre-existing particles [13].

*Streptomyces ghanaensis* ATCC14672 is famous for the production of Mm, a mixture of similar phosphoglycolipid antibiotics with a unique mechanism of action. Acting as the only known direct inhibitors of bacterial peptidoglycan glycosyltransferases—the enzymes involved in the penultimate step of cell wall biosynthesis—Mm have been considered to serve as the blueprint for the development of a novel class of antibacterials [14,15]. In addition to the very low amounts of Mm normally produced by *S. ghanaensis*, the strain also forms large mycelial particles growing in submerged cultures [15]. This forces the studies aimed to construct strains with higher Mm production titers and better growth characteristics. To achieve this, several heterologous hosts were successfully utilized to express the Mm biosynthetic gene cluster (*moe* cluster); however, the level of Mm biosynthesis was still lower than in the wild type [16,17]. Several studies have been undertaken to explore the regulation of Mm biosynthesis. Interestingly, regulatory genes are not present in the *moe* cluster and numerous global regulators were shown to influence Mm production [16,17,18,19,20,21,22,23,24,25,26]. A central role in the regulation of Mm biosynthesis belongs to the pleiotropic regulator AdpA_gh_, which was shown to directly activate the transcription of *moe* genes [19]. Orthologs of AdpA are omnipresent and highly conserved in *Streptomyces* and control the expression of numerous genes involved in morphogenesis and both primary and secondary metabolism [27,28]. Interestingly, a study in *Streptomyces coelicolor* also demonstrated a capacity of AdpA to inhibit the chromosome replication at the initial stage by binding to the replication origin (*oriC*), thus decreasing the access of the initiator protein (DnaA) [29]. Furthermore, deletion of *wblA*, a gene encoding a small Fe–S-containing cluster regulatory protein, drastically affects the antibiotic biosynthesis in many streptomycetes, including Mm production [18,24,30,31,32,33,34].

In this work, we applied genetic engineering approaches to construct a series of *S. ghanaensis* strains where the beneficial changes were sequentially combined, enabling the generation of a triple mutant strain with significantly improved growth particularities and Mm production titers. We showed that AdpA_gh_ plays an important role in the regulation of chromosome replication, thus crucially influencing the growth type of *S. ghanaensis*. Based on this observation, we created a mutant strain with deletion of amidotransferase *moeH5*, leading to accumulation of a significantly narrowed spectrum of Mm. Finally, we eliminated the negative regulator of Mm biosynthesis *wblA* to increasing antibiotic production levels. Overall, our findings set up a putative strategy for optimizing the natural products biosynthesis in other strains.

## 2. Materials and Methods

### 2.1. Bacterial Strains, Plasmids and Growth Conditions

Table S1 contains a list of bacterial strains and DNA constructs used in this work. *Escherichia coli* strains were grown in Luria-Bertani (LB) medium at 37 °C. Soya mannitol agar (SFM), oatmeal agar (OAT) and tryptic soy broth (TSB) liquid or agar (TSA) media were used to grow *S. ghanaensis*. Where necessary, the media were supplemented with appropriate antibiotics.

### 2.2. DNA Manipulation

Routine cloning manipulations were made in *E. coli* XL1-Blue according to standard procedures [35]. Polymerase chain reactions (PCR) were done using Phusion polymerase (ThermoFisher, Waltham, MA, USA) according to the manufacturer’s recommendations, with the set of primers listed in Table S2. Isolation of plasmid/chromosomal DNA was carried out following standard procedures [35]. Verification of DNA constructs was performed using DNA mapping with endonucleases of restriction, PCR and sequencing.

### 2.3. Intergeneric Conjugation with E. coli

*E. coli* ET12567 (pUZ8002) was used as a donor for conjugal transfer of plasmids into *S. ghanaensis* strains. Fresh recipient spore suspension was harvested from 7-day-old *S. ghanaensis* strains grown on OAT at 37 °C. After a 10 min heat shock at 50 °C, spores (4 × 10^7^ cfu) were mixed with *E. coli* cells (1 × 10^8^ cfu) carrying the different vectors and then plated on SFM supplemented with 60 mM CaCl_2_. Next, after 15 h of incubation at 28 or 37 °C (depending on a vector), plates were overlaid with 1 mL of water containing 1.6 mg of apramycin and 5 mg of phosphomycin. Usually, exconjugants appeared after 3 to 5 days of incubation. Nonsporulating mutant strains were first pre-grown in TSB for 24 h and then 1 mL of the resulting culture (~3 × 10^7^ cfu) was directly mixed with donor cells using the aforementioned procedure.

### 2.4. Microscopy

The mycelial morphology of *S. ghanaensis* in liquid-grown cultures was monitored using a Carl Zeiss Axiolab 1 microscope. For scanning electron microscopy (SEM), slices of *S. ghanaensis* lawns were cut off SFM agar plates and directly analyzed on a Quanta 250 environmental scanning electron microscope (ThermoFisher).

### 2.5. Generation of Mutant Strains

#### 2.5.1. Construction of *S. ghanaensis* O1

To construct a mutant strain, where AdpA_gh_bs in the *oriC* region of the *S. ghanaensis* chromosome were substituted to a nonsense sequence, the following scheme was used. Firstly, a 3-kb-long DNA fragment, consisting of AdpA_gh_bs plus two 1.5-kb-long regions of homology, was amplified from the *S. ghanaensis* chromosomal DNA by PCR using primers oriC_for/oriC_rev. The obtained amplicon was digested with XbaI and EcoRV and cloned into XbaI-EcoRV-cleaved pBluescriptKS+ to give pBloriC. In order to mutate AdpA_gh_bs, PCR mutagenesis was applied to amplify a 6 kb DNA fragment from pBloriC with primers oriC_mut_for/oriC_mut_rev. The obtained PCR fragment was first treated with T4 polynucleotide kinase and then self-ligated giving pBloriC-mut. Next, pBloriC-mut was cut with XbaI and EcoRV, and the resulting 3 kb DNA fragment was ligated into the respective sites of pKG1139, a vector based on a thermosensitive *pSG5* replicon carrying a reporter *gusA* gene [36]. The final plasmid pKGoriC-mut was conjugally transferred into *S. ghanaensis*. To promote an integration of pKGoriC-mut into the *S. ghanaensis* chromosome via homologous recombination, one exconjugant was inoculated in 15 mL of TSB containing apramycin. Five passages were conducted, where after each 24 h of growth at 40 °C (nonpermissive temperature for the *pSG5* replication), 10 μL of the culture was inoculated into a fresh medium with apramycin. Next, 1 mL of the resulting culture was plated on oatmeal agar. After seven days of incubation at 37 °C, serial dilutions of spore suspension were plated on SFM supplemented with 5-bromo-4-chloro-3-indolyl-D-glucuronide (X-Gluc) solution (50 μg mL^−1^). White colonies (indication of vector lost) were checked for the sensitivity to apramycin and then subjected to the PCR analysis. Substitution of AdpA_gh_bs was confirmed by sequencing, leading to the creation of *S. ghanaensis* O1.

#### 2.5.2. Construction of *S. ghanaensis* O1ΔH5

To delete *moeH5* from the *S. ghanaensis* O1 chromosome, a 6.4 kb DNA fragment comprising *moeH5* with two 2.4-kb-long regions of homology was PCR synthesized from the chromosome using primers moeH5_for/moeH5_rev. The resulting amplicon was ligated into pBluescriptKS+ cleaved with EcoRV to give pBlmoeH5. Next, PCR mutagenesis was used to remove the *moeH5* coding sequence. To do this, a pair of primers, moeH5_mut_f/moeH5_mut_r, was used to amplify a 7.8 kb DNA fragment comprising no *moeH5* gene from pBlmoeH5. Next, the PCR product was treated with T4 polynucleotide kinase and self-ligated, yielding pBlΔmoeH5. A 4.8 kb DNA fragment was cut out of pBlΔmoeH5 using XbaI-EcoRV and ligated into the respective sites of pKG1139, creating pKGΔmoeH5. Then, pKGΔmoeH5 was transferred to *S. ghanaensis* O1 by intergeneric conjugation with *E. coli*. Screening for double crossover mutants was done as described above, giving a mutant strain *S. ghanaensis* O1ΔH5.

#### 2.5.3. Construction of *S. ghanaensis* O1ΔH5ΔwblA

To generate a triple mutant with a *wblA* deletion, the analogous scheme was used. First, a 4.3 kb DNA fragment, consisting of *wblA* with two 2 kb flanking regions of homology, was amplified from the *S. ghanaensis* chromosome by PCR using the primers wblA_for/wblA_rev. The amplicon was ligated into pBluescriptKS+ cut with EcoRV to give pBlwblA. Then, PCR mutagenesis was done to generate a 6.9 kb fragment (containing no *wblA*) from pBlwblA with the primers wblA_mut_f/wblA_mut_r. It was subsequently treated with T4 polynucleotide kinase and self-ligated to create pBlΔwblA. A 4 kb XbaI-EcoRV cut DNA fragment was retrieved from pBlΔwblA and then cloned into pKG1139 digested with the respective enzymes to give pKGΔwblA. The final construct was conjugally transferred to *S. ghanaensis* O1ΔH5 and screening for the mutants was conducted as mentioned above. This resulted in construction of *S. ghanaensis* O1ΔH5ΔwblA.

### 2.6. Analysis of Mm Production

Cultivation of *S. ghanaensis* strains, Mm extraction and analysis of Mm production were performed according to the previously established protocols [17]. Routine quantification of Mm production levels was done on a UHPLC Thermo Fisher Scientific Ultimate 3000 SD system equipped with an automated liquid sampler, a diode array detector, and a TSQ Quantum Access MAX ESI mass spectrometer with a reversed-phase Nucleodur 100–5 C18ec column (Macherey-Nagel, 5 μm, 150 × 2 mm), as described before [24]. Four major compounds were monitored via LC-MS in *S. ghanaensis* WT extracts: MmA (MmA; *m/z* = 789.8 [M-2H]^2−^), nosokomycin B (NoB; *m/z* = 741.8 [M-2H]^2−^), moenomycin G (MmG; *m/z* = 770.8 [M-2H]^2−^) and desmethylated NoB (dNoB; *m/z* = 734.8 [M-2H]^2−^), whereas nosokomycin A (NoA; *m/z* = 742.3 [M-2H]^2−^) and desmethylated NoA (dNoA; *m/z* = 735.3 [M-2H]^2−^) were monitored in the strains carrying *moeH5* deletion.

For qualitative analysis of extracts from *S. ghanaensis*, a reversed-phased InfinityLab Poroshell 120 EC-C18 column (Agilent, 2.7 μm, 3.0 × 150 mm) was employed with the following mobile system: phase A—water and mobile phase B—MeCN, both with 0.5% acetic acid (vol/vol) as a solvent modifier. The solvents were delivered at 0.5 mL/min under a gradient elution program: 0 min 95% A, 0.5 min 95% A, 23 min 5% A, 26.5 min 5% A, 27 min 95% A, and 30 min 95% A. The mass spectrometer was operated in negative ESI mode using the parameters described earlier [24].

## 3. Results

### 3.1. Substitution of AdpA_gh_bs in oriC In Vivo Drastically Influences the Growth Particularities and Mm Production in S. ghanaensis

**Figure 1:**
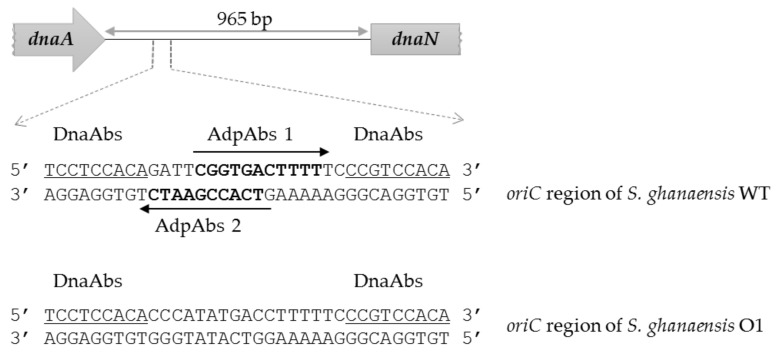
Schematic representation of the *S. ghanaensis oriC* region. Putative DnaA binding sites are underlined, whereas AdpAbs are in bold. The distance between the start and stop codons is shown.

In *S. ghanaensis* biosynthesis of Mm is strictly depended on the AdpA_gh_ activity, where it directly activates the transcription of key *moe* genes [19]. Moreover, in *S. coelicolor* AdpA binds to the *oriC* region, thus inhibiting chromosome replication [29]. Therefore, we aimed to evaluate whether substitution of AdpA_gh_bs in *oriC* influences growth and Mm production in *S. ghanaensis*. For this purpose, we first compared the *oriC* regions between *S. ghanaensis* and *S. coelicolor* for the presence of AdpAbs. As depicted in Figure 1, the nucleotide sequence of the *S. ghanaensis* DNA region, responsible for AdpA and DnaA binding, shares a high degree of similarity to that of *S. coelicolor* [29]. Particularly, there are two divergently oriented and partially overlapped putative AdpA_gh_bs (Figure 1). Analogous to *S. coelicolor*, they are located in the proximate vicinity of the DnaA binding sites (DnaAbs), indicating their involvement in the regulation of chromosome replication. It was shown that AdpA binding led to the extended protection of the DNA region and thus reduce accessibility of DnaAbs to the DnaA initiator protein [29]. Previous work on the *Streptomyces lividans* 66 *oriC* region suggested that replication of its chromosome depends on all DnaAbs, including those that surround AdpAbs [37] (Figure 1).

Therefore, to test the role of AdpA_gh_bs in the Mm production and growth of *S. ghanaensis*, we employed a PCR mutagenesis-based strategy (see Materials and Methods) to eliminate the overlapping AdpA_gh_bs 1 and 2 in vivo. We substituted them for a nonsense sequence in a way that the distance between two adjacent DnaAbs remained intact (Figure 1). The resulting *S. ghanaensis* strain carrying a mutation in both AdpA_gh_bs was named *S. ghanaensis* O1.

**Figure 2:**
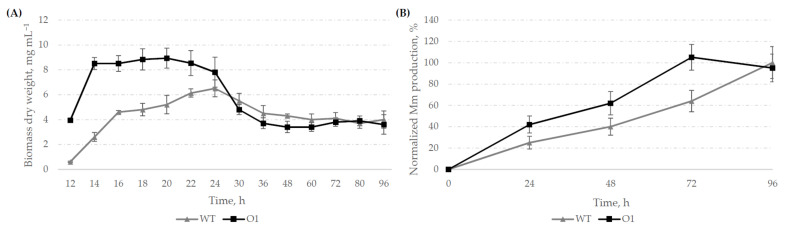
Substitution of AdpA_gh_bs in the *oriC* region severely affects the growth dynamic and Mm accumulation in *S. ghanaensis*. (**A**) Comparison of the biomass accumulation between the parental *S. ghanaensis* ATCC14672 (WT) and mutant *S. ghanaensis* O1 (O1) strains. (**B**) Dynamic of Mm production in *S. ghanaensis* ATCC14672 (WT) and *S. ghanaensis* O1 (O1). Error bars represent standard deviations. Amounts of Mm were normalized to equal amounts of biomass (dry weight) and were the mean values from at least three independent biological replicates. The mean value of the moenomycin mass peak area of the wild type detected after 96 h of fermentation was taken as 100%.

Next, we assessed changes in both Mm and biomass accumulation by *S. ghanaensis* O1 in comparison to the wild-type strain. Equal amounts of spores (1 × 10^7^ cfu) were used to initiate the fermentation and then the strains were cultivated in parallel in TSB for four days. Aliquots were collected from the growing cultures at certain time points and were subjected to further analysis. Biomass accumulation was used to monitor the growth dynamic. As shown in Figure 2A, on average, *S. ghanaensis* O1 accumulated two-fold more biomass during the first 20 h of growth compared to the wild type. Moreover, the mutant strain entered the stationary phase already after 14 h of growth, whereas it took 22–24 h to reach it for the wild type. After 30 h of cultivation, both strains displayed a similar growth pattern, indicating that mutation of AdpA_gh_bs strongly affects the type of growth, especially at early stages.

Following the growth dynamic, we evaluated the Mm production by *S. ghanaensis* strains. As depicted in Figure 2B, both analyzed strains accumulated relatively equal amounts of Mm after 96 h of cultivation, an established standard time period for Mm extraction in *S. ghanaensis* [17,19,24,25]. However, we observed a shift in the dynamic of antibiotic biosynthesis between strains. In comparison to the wild type, *S. ghanaensis* O1 produced a higher amount of Mm over the first three days of growth and reached its maximum after 72 h. After four days of cultivation, a concentration of Mm in biomass pellet slightly dropped down, most likely due to the cell lysis. In contrast, the control strain gradually accumulated the antibiotic over the entire fermentation time course and gained its highest titer after 96 h (Figure 2B). The obtained results suggest that binding of AdpA_gh_ to *oriC* strongly influences both growth pattern and time course of Mm biosynthesis in *S. ghanaensis*.

**Figure 3:**
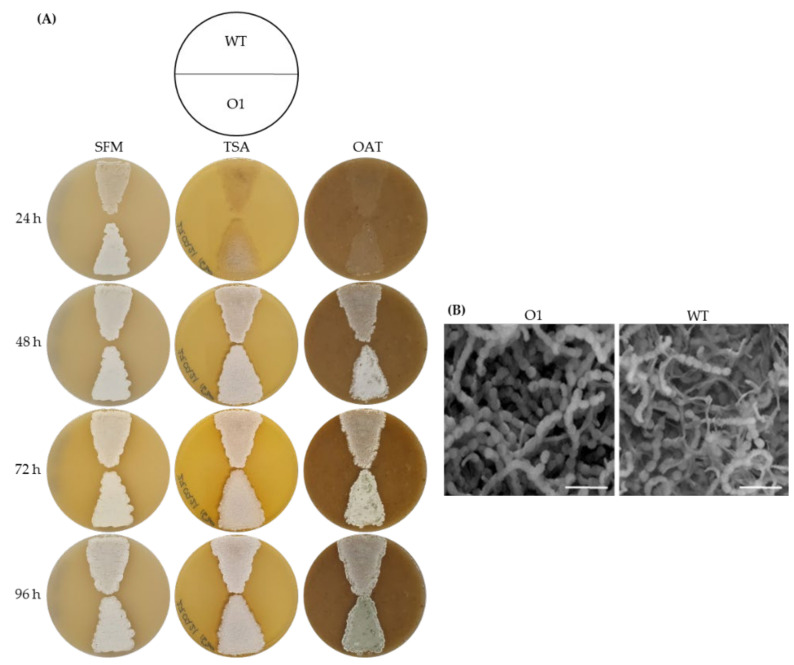
Substitution of AdpA_gh_bs in the *oriC* region has a little effect on morphological progression. (**A**) Phenotypes of *S. ghanaensis* ATCC14672 (WT) and *S. ghanaensis* O1 (O1) cultivated on SFM, TSA and OAT solid media at 37 °C. (**B**) More intensive sporulation observed in *S. ghanaensis* O1 (O1) in comparison to the wild type (WT) using SEM. Surfaces of the lawns from (**A**) grown on OAT for 96 h were used to obtain SEM images. Scale bars correspond to 5 μm.

Following our observations, we decided to check whether mutation of AdpA_gh_bs also affects the morphogenesis. For this purpose, two strains where grown in parallel on a series of media. Interestingly, no pronounced difference, either in growth dynamic or in morphological progression, was observed after four days of cultivation on solid media (Figure 3A). However, after four days of growth, scanning electron microscopy revealed a slightly intensive and denser sporulation in *S. ghanaensis* O1 (Figure 3B). To investigate this, we calculated a titer of spores formed by the two analyzed strains after four days of incubation on OAT. Indeed, in comparison to the wild type, the mutant strain produced on average 25% more spores per plate.

**Figure 4:**
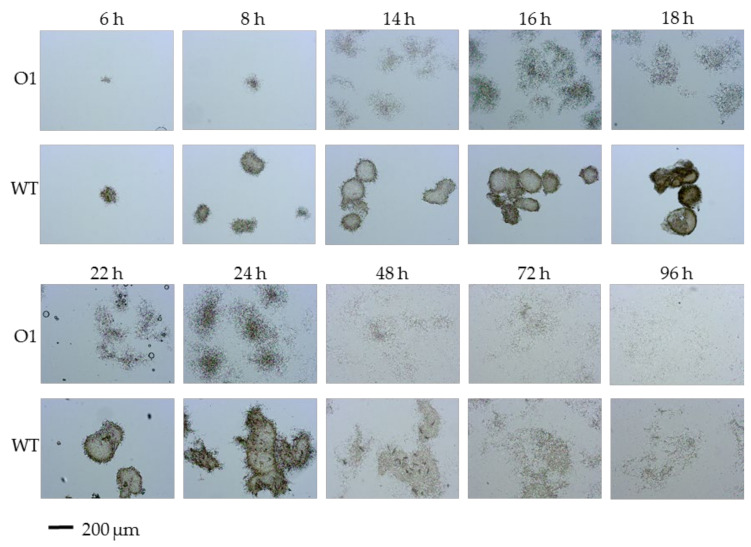
Liquid-culture morphology of the *S. ghanaensis* ATCC14672 (WT) and *S. ghanaensis* O1 (O1) strains grown in TSB for 96 h. The scale bar is 200 μm.

Since the most distinct changes in growth particularities were detected during the cultivation of *S. ghanaensis* strains in liquid media, we examined the mycelial morphology of strains grown in TSB. The *S. ghanaensis* wild-type strain typically grows forming large mycelial pellets (Figure S1), which is disadvantage, as such a growth type results in slow nutrient utilization and hampers routine laboratory manipulations. To compare differences in growth dynamic, an equal amount of seeding material (spores, 1 × 10^7^ cfu) was inoculated into TSB. Sample aliquots were collected from growing cultures and then mycelial morphology was directly analyzed. The beginning of aggregation of the germinated spores was already noticeable after 2 h of growth in the wild type, whereas there was a 2 h delay in *S. ghanaensis* O1 (data not shown). After 6 h of growth both strains formed mycelial structures; however, they were bigger and denser in the parental strain (Figure 4). The difference in type of growth became more profound over the time of cultivation. Thus, after 14 h since the inoculation, individual pellets produced by the wild type started to aggregate, leading to the formation of large mycelial complexes (Figure 4). This tendency continued for the first two days of growth and then the dynamics of pellet fragmentation started to prevail over aggregation. This led to a more dispersed pellet morphology, especially after 48 h of growth, whereas the mutant strain formed almost no mycelial pellets over the entire time course of cultivation, except upon increasing the biomass density patches of the dispersed mycelia entangled in open pellet-like structures (Figure 4).

The dispersed growth of *S. ghanaensis* O1, an accelerated growth rate, a shorter duration of fermentation along with an increased Mm accumulation are clearly beneficial over the parental strain. This set up a starting point for further manipulations to improving the Mm production in *S. ghanaensis*.

### 3.2. A Marker- and “Scar”-less Deletion of Amidotransferase Gene moeH5 Narrows down the Spectrum of Mm Produced by S. ghanaensis O1ΔH5

**Figure 5:**
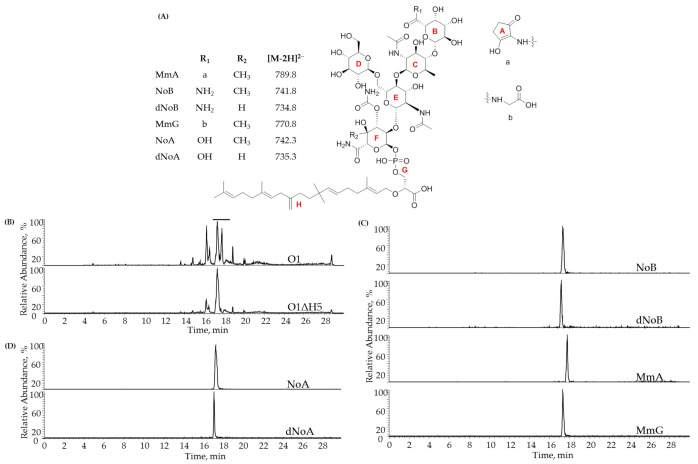
Deletion of the amidotransferase gene *moeH5* significantly narrows down the spectrum of Mm produced by *S. ghanaensis* O1ΔH5. (**A**) Structures of Mm related to this work. (**B**) LC-MS (TIC) analysis of the methanol extracts from biomass of *S. ghanaensis* O1 (O1) and *S. ghanaensis* O1ΔH5 (O1ΔH5). Thick blue line corresponds to the Mm retention time. (**C**) MS extracted ion chromatograms corresponding to main Mm derivatives accumulated in *S. ghanaensis* O1. (**D**) The strongly reduced spectrum of Mm produced by *S. ghanaensis* O1ΔH5. Shown are MS extracted ion chromatograms corresponding to NoA and dNoA.

A complex of Mm produced by *S. ghanaensis* includes numerous derivatives of MmA, with minor modifications [15]. This not only interferes with the purification of desired compounds but also significantly hinders the quantification of antibiotic production during routine lab manipulations. The structural heterogeneity of Mm in *S. ghanaensis* is originated from several variables [15]. The majority of Mm contains different portions at the carboxylic acid group of galactopyranuronic acid unit B (Figure 5A). It can be linked either to a 2-aminocyclopentane-1,3-dione (C5N) chromophore unit A, or converted into a carboxamide, or extended with glycine, serine, cysteine or alanine. Recently it was shown that the greatest natural diversity in the Mm family of antibiotics is mediated by the amidotransferase MoeH5, which possesses a broad substrate specificity [38]. To reduce a spectrum of Mm typically produced by the wild-type strain, we decided to eliminate *moeH5* from the chromosome of *S. ghanaensis* O1. To construct a marker- and “scar”-free strain, we employed a PCR mutagenesis strategy. A *moeH5* knockout plasmid was created in a way that allows a “clear” deletion of the entire ORF. Since an upstream gene *moeGT1* overlaps with *moeH5*, only the start, one following and the stop codons remained in the *S. ghanaensis* O1ΔH5 chromosome to keep in frame the DNA sequence.

To compare metabolic profiles of *S. ghanaensis* O1ΔH5 and O1, both strains were cultivated in TSB for four days. Methanol extracts from the biomass were directly analyzed via UHPLC-MS (Figure 5B). As shown in Figure 5C, in *S. ghanaensis* O1 the most abundant peaks were negatively double charged ions corresponding to NoB (m/z = 741.8), MmA (m/z = 789.8), dNoB (m/z = 734.8) and MmG (m/z = 770.8). A contribution of other Mm to the total antibiotic production in *S. ghanaensis* O1 was relatively low. In contrast to the parental strain, deletion of *moeH5* greatly narrowed down Mm spectrum in *S. ghanaensis* O1ΔH5 (Figure 5D). The mutant almost exclusively accumulated NoA (m/z = 742.3), with a minor fraction of dNoA (m/z = 735.3). It is worth noting that there were no obvious changes, either in the growth dynamic or in the timing of morphogenesis between the analyzed strains.

In summary, we constructed the mutant *S. ghanaensis* O1ΔH5 that produces a significantly narrowed spectrum of antibiotics. Additionally, deletion of *moeH5* did not introduce any extra DNA (e.g., antibiotic resistance markers, recombinase recognition sites, etc.) in the chromosome of the producing strain, making it easily susceptible for the next genetic manipulations.

### 3.3. Deletion of the Regulatory Gene wblA_gh_ Strongly Improves NoA Production in S. ghanaensis O1ΔH5ΔwblA

Unlike the majority of antibiotic biosynthetic gene clusters, in *S. ghanaensis*, Mm biosynthesis includes no cluster-situated regulators involved in the regulation of metabolic pathway [14,17,19]. This is disadvantageous due to the fact that routine approaches (e.g., overexpression of positive or deletion of negative cluster-situated regulators) are not applicable for improving of Mm production titers. Many studies have identified numerous global pleiotropic regulators governing Mm production. One of them—*wblA*—a gene encoding a Fe–S cluster-containing transcription factor, involved in the late stages of morphological differentiation, was shown to negatively operate Mm synthesis in *S. ghanaensis* [18,24]. To improve NoA accumulation by *S. ghanaensis* O1ΔH5, we created a triple mutant where *wblA_gh_* was deleted from the chromosome via homologous recombination. The gene knockout was performed in the same way as described above, leading to the construction of a marker- and “scar”-less mutant *S. ghanaensis* O1ΔH5ΔwblA.

**Figure 6:**
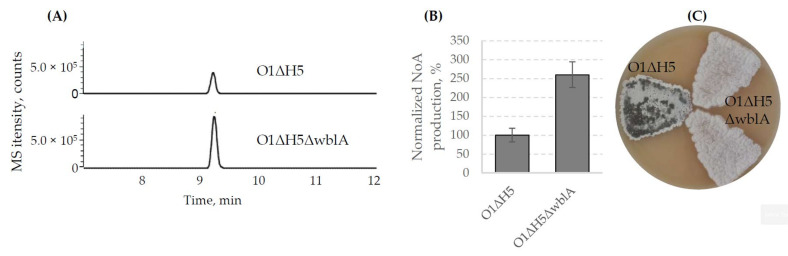
Deletion of *wblA_gh_* drastically affects both NoA accumulation and morphological progression. (**A**) LC-MS analysis of methanol extracts from biomass of *S. ghanaensis* O1ΔH5 (O1ΔH5) and *S. ghanaensis* O1ΔH5ΔwblA (O1ΔH5ΔwblA). Shown are MS extracted ion chromatograms corresponding to NoA + dNoA. (**B**) NoA + dNoA production titers by *S. ghanaensis* strain as determined by LC-MS. (**C**) Inability of *S. ghanaensis* O1ΔH5ΔwblA to produce spores even after prolonged incubation. Strains were grown for 7 days on MS.

To compare the production titers of NoA, *S. ghanaensis* O1ΔH5ΔwblA and O1ΔH5 were grown in TSB for four days. Extracts from their biomass were subjected to analysis via LC-MS. As depicted in Figure 6A, deletion of *wblA_gh_* significantly improved antibiotic production in O1ΔH5ΔwblA compared to the parental strain. On average, the *wblA_gh_*-deficient mutant accumulated 2.6-fold more NoA than the O1ΔH5 strain (Figure 6B). In addition, elimination of *wblA_gh_* strongly impaired the morphological progression, as the mutant strain failed to sporulate. In comparison to the control strain, even after prolonged incubation on solid media, the surface of *S. ghanaensis* O1ΔH5ΔwblA remained white, indicating an inability to produce a green spore pigment associated with mature spores (Figure 6C).

### 3.4. Comparison of Conjugal Efficacy between E. coli and the Generated Mutant Strains

Efficiency of DNA introduction into *Streptomyces* cells is one of the key factors required for the successful genetic manipulations. Among all the available ways of DNA transfer in the genetics of streptomycetes, *E. coli* – *Streptomyces* intergeneric conjugation is the most widely used method due to its relative simplicity and high efficiency [39]. Since some of our genetic manipulations drastically changed both the morphology and growth dynamic, we aimed to compare the efficiency of DNA introduction between the generated *S. ghanaensis* strains. To achieve that, we used two of the most common actinobacterial vectors broadly used in *S. ghanaensis* [14,24,25]: an integrative φC31-based pSET152 and an oligocopy replicative pKC1139 [39]. It was shown that in *S. ghanaensis*, pSET152 integrates into *attB*^φC31^ only once per genome [40], whereas pKC1139 normally replicates at the temperatures lower than 30 °C due to the presence of a thermosensitive pSG5 replicon that restricts replication at nonpermissive conditions (t > 36 °C) [41].

The pSET152^+^ and pKC1139^+^ exconjugants of the *S. ghanaensis* wild type were obtained at a 2.5- and 3-fold lower frequency, respectively, in comparison to *S. ghanaensis* O1 (Table 1). Next, we tested the exconjugants occurrence using *S. ghanaensis* O1ΔH5 as a recipient. Predictably, there was a little difference in conjugal frequency for both tested vectors with respect to its parental strain *S. ghanaensis* O1, since elimination of *moeH5* had no influence on the growth characteristics. Lastly, we determined the influence of *wblA_gh_* deletion on the conjugal efficiency. The appearance of exconjugants drastically decreased by 6- and 7-fold for pSET152 and pKC1139, respectively, compared to the parental strain (Table 1).

In summary, elimination of AdpA_gh_bs in the *oriC* region strongly improved the efficacy of conjugal transfer using both replicative and integrative vectors. In contrast, deletion of *wblA_gh_*, which led to the repression of sporulation in *S. ghanaensis*, significantly reduced the frequency of exconjugants occurrence.

## 4. Discussion

The worldwide spread of multidrug-resistant pathogens is a rising global problem. Uncontrolled and unreasonable administration of antibacterials over the last several decades have led to the accumulation of superbugs—microorganisms that have developed the ability to resist against commonly prescribed medications. The growing number of superbugs resistant to the cell-wall inhibitor vancomycin is one of the most urgent calls for public health, as this causes a number of deaths around the world annually [42]. Nowadays, novel antibiotics, able to combat multidrug-resistant pathogens, are urgently needed. The introduction of new classes of antibiotics in clinical applications has been limited over the last 40 years because of several reasons [43]. Despite the huge portfolio of biosynthetic gene clusters encoded by actinobacterial genomes, only a small number of NPs are abundantly produced during the laboratory conditions [44]. Many novel techniques have been applied to boost the expression of poorly expressed clusters; however, in most of the cases it tends to rediscovery of known antibiotics [45,46,47,48,49]. This reinforces an interest in promising antibiotics that were discovered many years ago.

Mm belong to a small family of phosphoglycolipid antibiotics that were initially discovered in 1965 [50,51]. Mm inhibits the cell wall biosynthesis in unique way by direct binding to bacterial transglycosylases—the enzymes involved in the last steps of cell wall assembly. In comparison to the clinically used glycopeptide antibiotic vancomycin, MmA is up to 1000-fold more potent against various Gram-positive bacteria, including vancomycin-resistant pathogens [52]. Despite their high potency, Mm have never been introduced into clinical practice because of their suboptimal pharmacokinetic properties. It is thought to be mainly due to the presence of the C_25_ carbon chain, leading to their long half-life in the blood stream [53,54]. Therefore, several studies have been initiated to construct Mm analogs with improved pharmacokinetics employing both chemoenzymatic and genetic engineering techniques [15]. Numerous derivatives with altered biological activities were created, using the active Mm pharmacophore as a starting point [55,56,57,58]. Taking into account that chemical synthesis of Mm is challenging [59], the production of novel Mm derivatives through fermentation and/or chemoenzymatic approaches would be highly desirable. Nevertheless, the Mm complex has been widely used as animal growth promoters for cattle, swine and poultry [60]. Strikingly, no profound impact on the Mm antimicrobial resistance due to an intensive use of Mm in animal feeding has been observed [54,61].

*S. ghanaensis* is one of few streptomycetes known for the ability to produce Mm. Many studies have been conducted in order to improve low Mm production levels, including utilization of heterologous hosts for the expression of *moe* genes [14,16,17,19,23]. However, none of the tested hosts were superior over the wild-type strain in terms of Mm productivity. Thus, genetic and metabolic engineering of the native producer would be the logical continuation of the work to construct overproducing strains. However, when grown in submerged cultures, *S. ghanaensis* typically forms large mycelial pellets. This type of growth is disadvantageous from a biotechnological point of view and it complicates routine lab manipulations. In this work, we demonstrate the pivotal role of AdpA_gh_ in controlling the *S. ghanaensis* growth type. Substitution of AdpA_gh_bs to a nonsense sequence in the *oriC* region drastically changed the growth characteristics of the mutant strain. In comparison to the wild type, *S. ghanaensis* O1 grew in a very dispersed way, forming no mycelial clamps over the entire fermentation time course. Moreover, the mutant accumulated biomass much faster than the parental strain, indicating the deregulated chromosome replication and therefore increased cell division rate. As was previously described in *S. coelicolor*, with the time of growth in mycelial compartments AdpA reaches its highest levels [62], which leads to the inhibition of chromosome replication. Most likely, over-replication in *S. ghanaensis* O1 interferes with the processes involved in aggregation of hyphae, avoiding the formation of dense mycelial particles common for the wild-type strain. For example, in *S. lividans* there are several key factors participating in complex mechanisms of pellet formation. *S. lividans* mutants deficient in *mat* genes, which encode putative polysaccharide synthases involved in hyphae aggregation, form very small and open mycelia [12]. It is worth noting that in many streptomycetes overexpression of the cell-division activator protein SsgA had a major effect on mycelial morphology in submerged cultures, leading to the fragmented growth of mycelia [5]. Interestingly, in *S. lividans* and *S. coelicolor* the cellulose synthase-like protein CslA is responsible for an extracellular polysaccharide that determines the pellet morphology, with a dispersed morphology of *cslA* null mutants [63]. At the moment, we cannot fully explain the detailed mechanisms leading to the dispersed growth of *S. ghanaensis* O1. Whatever the real mechanism is, it is useful practically because an improved dispersed growth of strains is one of the key requirements for industrial utilization. Curiously, the very recent data provided by Płachetka et al. [64] have shown that the AdpA ortholog in *Streptomyces venezuelae* does not interact with its *oriC* region. Likely, this is one of the main factors explaining the dispersed growth common for *S. venezuelae*. Bioinformatical analysis of many streptomycetes genomes identified the presence of AdpAbs in *oriC* regions [29], suggesting that such mechanisms are omnipresent across *Streptomyces* spp. This fact points to the versatile genetic engineering strategy for improving the growth particularities of streptomycetes, producers of commercially important NPs.

Common for NP biosynthetic pathways, there are few final compounds accumulated during the fermentation course [65,66,67]. This strongly influences the industrial utilization of the producing microorganism, since it requires the expensive and complex rounds of purification to separate the desired molecule. Analogously, a complex of Mm produced by *S. ghanaensis* comprises a number of compounds with a slightly modified structure [15]. The three most abundant metabolites were MmA, NoB and MmG, which were almost equally accumulated and comprised the major fraction of Mm produced by the wild type under the tested conditions. Tremendous structural heterogeneity of many Mm is determined by the activity of amidotransferase MoeH5. Due to the broad substrate specificity, MoeH5 can introduce various modification to the “core” molecule NoA [38]. In this work, we employed a PCR mutagenesis-based strategy to eliminate *moeH5* from the chromosome of producing strain. The used gene knockout system allows constructing marker- and “scar”-less deletions without introducing extra foreign DNA. In contrast to the well-known and widely used marker removal systems that utilize site-specific recombinases (e.g., Cre, Dre, Flp) [68,69], leaving a short oligonucleotide region called “scar” that may limit further experiments [70], it permits creation of engineered strains with a virtually unlimited number of modifications. That is especially useful for the large genome optimization projects involving multiple genetic manipulations. The resulting double knockout mutant *S. ghanaensis* O1ΔH5 accumulated the growth benefits of the parental *S. ghanaensis* O1 strain and produced a significantly narrowed spectrum of Mm accumulating almost exclusively NoA. The strain may be used as a background for the chemoenzymatic synthesis of Mm analogues with altered biological activities.

Biosynthesis of secondary metabolites in streptomycetes undergoes a fine-tune regulation at multiple levels. Usually, natural producers accumulate a relatively low amount of desired compound and antibiotic titer improvement is the main requirement for the industry. *S. ghanaensis* synthesizes Mm in milligram quantities, which has forced numerous investigations aimed to improving production levels. Because of the absence of cluster-situated regulators associated with *moe* genes, improvement of Mm biosynthesis is challenging. Some global regulators were shown to be involved in governing of Mm production in streptomycetes. In this study, we deleted the regulatory gene *wblA_gh_* from the chromosome of *S. ghanaensis* O1ΔH5 using the aforementioned marker- and “scar”-less technique. The *wblA* orthologs are conserved across the *Streptomyces* genus and many studies, including that for Mm synthesis, reported them as negative regulators of antibiotic production [31,32,33]. Deletion of *wblA_gh_* strongly increased NoA accumulation by the resulting triple mutant strain *S. ghanaensis* O1ΔH5ΔwblA, while completely blocking the morphogenesis at the aerial mycelium level.

Successful genome engineering relies on an efficient and relatively easy way of DNA transfer into streptomycetes. Therefore, we evaluated the influence of applied genetic manipulations on the efficacy of DNA transfer in intergeneric conjugation between *E. coli* and *S. ghanaensis*. Interestingly, we observed a profound impact of substitution of AdpA_gh_bs in the *oriC* region on the conjugal efficiency. In comparison to the wild-type strain, both pSET152 and pKC1139 were transferred with 2.5–3-fold higher frequency using *S. ghanaensis* O1 as a recipient. Likely, the way that the mutant grows is the most plausible explanation of the increased conjugal ability. In contrast to the parental strain, which forms dense and large mycelial particles that would limit the physical contacts with donor cells, *S. ghanaensis* O1 produces almost no mycelial pellets, establishing an efficient and close contact between interacting cells. As anticipated, deletion of amidotransferase *moeH5* did not influence the morphological progression and resulted in no changes in the exconjugants occurrence compared to *S. ghanaensis* O1. Lastly, the frequency of conjugation was greatly reduced when *S. ghanaensis* O1ΔH5ΔwblA was used as a recipient strain. This perfectly correlates with the inability of the *wblA_gh_*-defficient mutant to produce spores. Even though we used an equal amount of recipient cells (~10^7^ cfu), the exconjugants occurrence was decreased more than 6-fold when compared to *S. ghanaensis* O1ΔH5. In many tested cases, the conjugal efficiency was shown to be much higher when a spore suspension rather than mycelium was used [39,71,72]. Therefore, despite the increased NoA production in *S. ghanaensis* O1ΔH5ΔwblA, its further utilization might be limited in experiments demanding a high efficiency of conjugation.

## 5. Conclusions

In this work, we employed genome engineering approaches to construct *S. ghanaensis* strains with improved growth and antibiotic production characteristics. First, we in vivo substituted AdpA_gh_bs in the *oriC* region in the *S. ghanaensis* chromosome. Strikingly, in contrast to the wild type, the mutant *S. ghanaensis* O1 strain grew in a dispersed way, forming no mycelial pellets and displaying an accelerated growth rate. Moreover, the maximum Mm biosynthesis was observed 24 h in advance in the mutant compared to the parental strain. Next, a marker- and “scar”-less elimination of the amidotransferase gene *moeH5* greatly reduced the spectrum of Mm produced by *S. ghanaensis* O1ΔH5, leading to the accumulation of almost exclusively NoA. Additionally, knockout of the negative regulator of antibiotic production *wblA_gh_* strongly improved the NoA biosynthetic titers but resulted in a strain deficient in sporulation. Finally, we compared the efficiency of DNA transfer using intergeneric conjugation with *E. coli*. We observed the strong beneficial effect of the AdpA_gh_bs substitution on the frequency of exconjugants occurrence, whereas deletion of *wblA_gh_* drastically reduced the conjugal efficacy. Overall, we constructed a triple mutant carrying no extra foreign DNA, whose type and accelerated growth, narrowed spectrum and increased level of antibiotic synthesis are clearly supreme over the original strain. The technology described in this paper can be useful background information for further projects focused on production titers optimization in other strains.

## Figures and Tables

**Table 1:** Frequency of exconjugants occurrence in the *S. ghanasensis* strains.

Strain	pSET152	pKC1139
*S. ghanaensis* ATCC14672	(4 ± 0.3) × 10^−5^	(5 ± 0.2) × 10^−6^
*S. ghanaensis* O1	(1 ± 0.1) × 10^−4^	(1.5 ± 0.2) × 10^−5^
*S. ghanaensis* O1ΔH5	(9 ± 0.8) × 10^−5^	(1.4 ± 0.3) × 10^−5^
*S. ghanaensis* O1ΔH5ΔwblA	(1.6 ± 0.25) × 10^−5^	(2.1 ± 0.4) × 10^−6^

## Data Availability

The data presented in this study are available in this article and Supplementary Materials.

## Supplementary Materials

The following supporting information can be downloaded at: https://www.mdpi.com/article/10.3390/microorganisms10010030/s1, Figure S1: Morphology of the *S. ghanaensis* strains grown in TSB for 24 h. Table S1: Strains and plasmids used in this work. Table S2: Primers used in this work.
