# Supplementary material for: Genetic Engineering of Streptomyces ghanaensis ATCC14672 for Improved Production of Moenomycins
Source: Microorganisms. 2021 Dec 24;10(1):30. doi: 10.3390/microorganisms10010030 (PMC8778134; doi:10.3390/microorganisms10010030)
Supplement: Supplementary file 1 [file microorganisms-10-00030-s001.zip › microorganisms-1494032-supplementary.pdf]

microorganisms

Supplementary Materials

Table S1. Strains and plasmids used in this work.

Strain/plasmid	Description/Function	Source
Strains		
S. ghanaensis ATCC14672	Wild-type (WT) moenomycin producer	ATCC
S. ghanaensis O1	WT derivative, $AdpA_{gh}$bs replacement in *oriC* region	This work
S. ghanaensis O1ΔH5	*S. ghanaensis* O1 derivative, deletion of *moeH5*	This work
S. ghanaensis O1ΔH5ΔwblA	*S. ghanaensis* O1ΔH5 derivative, deletion of *wblA*	This work
E. coli XL1Blue	Host strain for DNA cloning	Agilent
E. coli ET12567 (pUZ8002)	Host for *E. coli*-streptomycetes conjugation	[1]
Plasmids		
pBluesctiptIIKS+	Cloning vector, Ap^R	Addgene
pSET152	φC31-based integrative vector, Am^R	[2]
pKC1139	*Streptomyces pSG5*-based oligocopy vector, Am^R	[3]
pKG1139	pKC1139-based vector with *gusA* reporter gene, Am^R	[4]
pBloriC	pBluescriptIIKS+ carrying a 3 kb *oriC* region	This work
pBloriC-mut	pBloriC carrying a substitution of $AdpA_{gh}$bs	This work
pKGoriC-mut	pKG1139 carrying a XbaI-EcoRV fragment for $AdpA_{gh}$bs replacement	This work
pBlmoeH5	pBluescriptIIKS+ carrying *moeH5* with 2.4 kb flanks	This work
pBlΔmoeH5	pBlmoeH5 with deleted *moeH5*	This work
pKGΔmoeH5	pKG1139 based construct for deletion of *moeH5*	This work
pBlwblA	pBluescriptIIKS+ carrying *wblA* with 2 kb flanks	This work
pBlΔwblA	pBlwblA with deleted *wblA*	This work
pKGΔwblA	pKG1139 based construct for deletion of *wblA*	This work

Table S2. Primers used in this work.

Primers	Sequence
oriC_for	aaatctagaCCGGACTACCGTCCGCAG
oriC_rev	aaagatatcCGGTCGGGAAGAGCGTG
oriC_mut_for	atgacCTTTTTCCCGTCCACACCCTG
oriC_mut_rev	atgggTGTGGAGGACGGCCGTAAC
moeH5_for	aaatctagaGATGACCAGCACCGACC
moeH5_rev	aaagatatcGAAGCACACGTAGCCGTC
moeH5_mut_f	tagGCAGACCCCCACTTCACG
moeH5_mut_r	CGTCATCGGGCGTCCAG
wblA_for	aaatctagaCGTTGCCCTGGACCACG
wblA_rev	aaagatatcCCGAGGAGTACGCCGAGC
wblA_mut_f	TGAGGGTTCCTCAGGGGTC
wblA_mut_r	TTCATCCGGATCGGTAGTGC

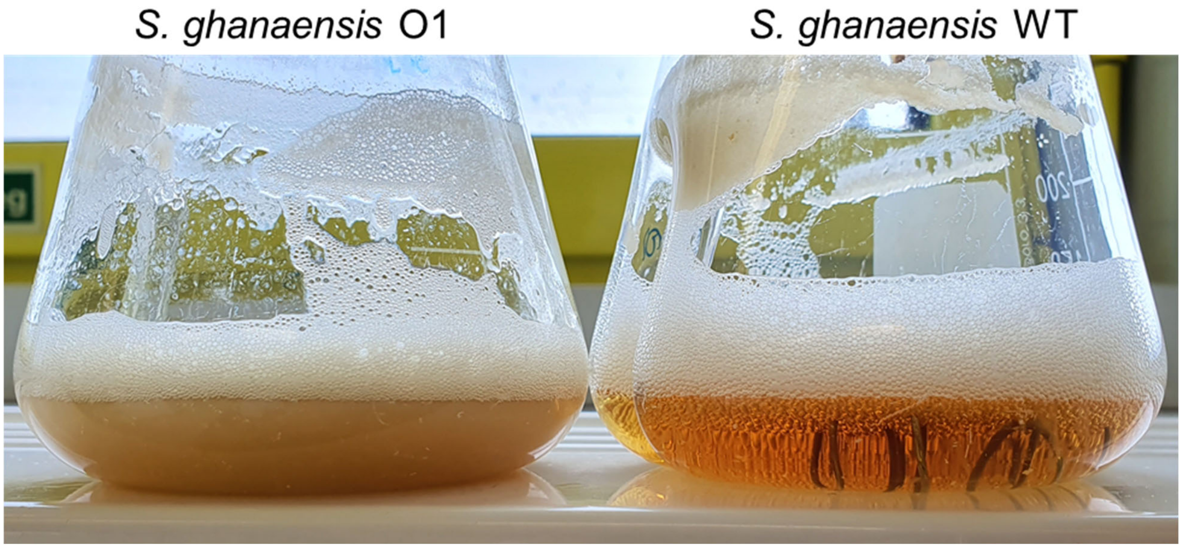

Figure S1. Morphology of the *S. ghanaensis* strains grown in TSB for 24 h.

References

1. Kieser, T. *Practical streptomyces genetics;* John Innes Foundation: Norwich, 2000, ISBN 0-7084-0623-8.
2. Bierman, M.; Logan, R.; O'Brien, K.; Seno, E.T.; Nagaraja Rao, R.; Schoner, B.E. Plasmid cloning vectors for the conjugal transfer of DNA from Escherichia coli to Streptomyces spp. *Gene* **1992**, *116*, 43–49, doi:10.1016/0378-1119(92)90627-2.
3. Muth, G. The pSG5-based thermosensitive vector family for genome editing and gene expression in actinomycetes. *Appl. Microbiol. Biotechnol.* **2018**, *102*, 9067–9080, doi:10.1007/s00253-018-9334-5.
4. Myronovskyi, M.; Welle, E.; Fedorenko, V.; Luzhetskyy, A. Beta-glucuronidase as a sensitive and versatile reporter in actinomycetes. *Appl. Environ. Microbiol.* **2011**, *77*, 5370–5383, doi:10.1128/AEM.00434-11.
